# Seroprevalence, Associated Factors, and Molecular Detection of *Toxoplasma gondii* Infection in Brazilian Gold Miners Working Informally in French Guiana

**DOI:** 10.1155/jotm/5932796

**Published:** 2026-04-15

**Authors:** Lucas Almeida Zangirolami, Igor Falco Arruda, Raíssa Cristina Ferreira Ramos, Fernanda da Silva Lopes, Caroline Martins da Costa, Maylis Douine, Amanda Figueira da Silva, Alice Sanna, Muriel Galindo, Yann Lambert, Martha Cecília Suárez-Mutis, Maria Regina Reis Amendoeira

**Affiliations:** ^1^ Protozoology Laboratory, Oswaldo Cruz Institute, Oswaldo Cruz Foundation, Rio de Janeiro, Brazil, fiocruz.br; ^2^ Graduate Program in Tropical Medicine, Oswaldo Cruz Institute, Oswaldo Cruz Foundation, Rio de Janeiro, Brazil, fiocruz.br; ^3^ Department of Microbiology and Parasitology, Laboratory of Innovations in Communication, Inclusion, and Popularization of Parasitology, Biomedical Institute, Fluminense Federal University, Niterói, Rio de Janeiro, Brazil, uff.br; ^4^ Inserm Clinical Research Center, Cayenne Hospital Center, Cayenne, French Guiana; ^5^ Laboratory of Parasitic Diseases, Oswaldo Cruz Institute, Oswaldo Cruz Foundation, Rio de Janeiro, Brazil, fiocruz.br

**Keywords:** Amazon, artisanal and small-scale gold mining, toxoplasmosis

## Abstract

*Toxoplasma gondii* is the etiologic agent of toxoplasmosis, a widely distributed zoonosis with relevance in human and veterinary medicine. The aim of this study was to assess the prevalence of *T. gondii* infection in Brazilian miners working informally in French Guiana. To this end, 480 individuals were recruited from resting places in the municipality of Oiapoque, Amapá, Brazil. Whole blood samples on filter paper were analyzed for IgM and IgG antibodies by ELISA. Nested PCR of the GRA7 gene was used to detect parasite DNA. Sociodemographic information, characteristics of the mining sites, and lifestyle data were collected using epidemiological questionnaires analyzed using univariate and multivariable models. The results showed that 79.3% of the individuals were IgG seropositive, while 0.8% were IgM seropositive. These were also IgG seropositive. In addition, *T. gondii* DNA was detected in 2% of the samples analyzed by nPCR, all of which were IgG seropositive and none of which were IgM seropositive. The high seropositivity observed, without association with sociodemographic or occupational variables, suggests that *T. gondii* exposure may be driven by environmental conditions related to Amazonian mining. These findings highlight the need for further investigation of environmental transmission sources and support targeted public health interventions, including improved water safety, sanitation, surveillance, and health education tailored to mining communities.

## 1. Introduction

*Toxoplasma gondii* is a facultative heteroxenous intracellular protozoan that has members of the Felidae family, including the domestic cat, as definitive hosts, and birds and mammals as intermediate hosts [1]. This parasite is the causative agent of toxoplasmosis, a zoonotic protozoosis responsible for significant human and animal health issues worldwide [2]. Human transmission occurs through the ingestion of water and food contaminated with sporulated oocysts, through the consumption of raw or undercooked meat containing tissue cysts, and through transplacental transmission of tachyzoites [3]. In Brazil, *T. gondii* infection exhibits a high prevalence, particularly in the Amazon region [4–6].

In French Guiana, there are no studies on the prevalence of anti‐*T. gondii* antibodies in the general population. However, toxoplasmosis outbreaks have been described in remote communities along the Maroni River, on the border with Suriname, and along the Oiapoque River, on the border with Brazil [7–9]. Over the past decades, numerous cases of severe acute toxoplasmosis involving immunocompetent individuals have been reported in French Guiana, associated with forest activities, [10–13]. These observations indicate that potential risk factors associated with the sylvatic cycle of *T. gondii* may be related to infections in individuals who venture into the forest, such as artisanal and small‐scale Brazilian gold miners.

Illegal mining in the Amazon is responsible for environmental damage related to deforestation, mercury contamination, and threats to traditional communities, as well as posing risks for the acquisition and spread of zoonotic diseases, such as *T. gondii* infection [14, 15]. In the Guiana Shield, the handling and consumption of game meat and the drinking of surface water have already been pointed out as possible routes of transmission of this protozoan [9, 11]. The artisanal and small‐scale gold miners represent a population of interest because they make incursions to isolated locations on the French side of the Amazon rainforest, where the gold extraction sites are located [16]. The estimated number of miners working in French Guiana is around 10,000, mostly Brazilians, who, despite spending more time in isolated forest areas, return to resting places on the Brazilian side to buy supplies, for leisure, and for medical visits [17].

The poor hygiene conditions and eating habits of this population can also increase the risk of acquiring parasitic infections. Douine et al. [18] showed that among the miners working in French Guiana, 95.4% did not have access to drinking water, with wells and rivers as their main sources of water, and two‐thirds reported consuming game meat an average of five times in a 30‐day period prior to the interview. In addition, 17.4% of the individuals mentioned the presence of domestic cats on the mining site. Given this scenario, the authors reinforce the need to investigate *T. gondii* infection in the population of artisanal and small‐scale gold miners, as well as other diseases [19, 20]. Considering this background, this study aims to investigate the prevalence by serological and molecular tools and associated factors of *T. gondii* infection among Brazilian gold miners working informally in French Guiana.

## 2. Methodology

### 2.1. Location and Type of Study

**FIGURE 1:**
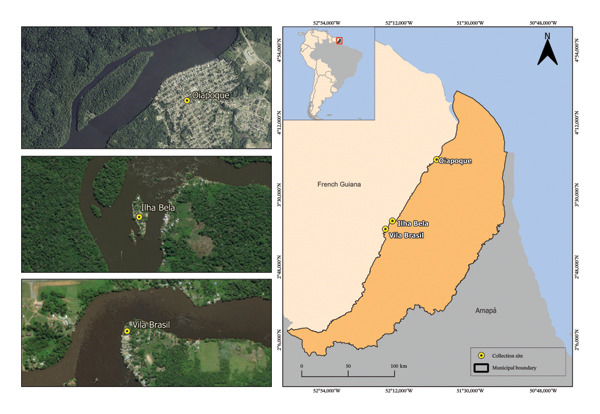
Map showing the collection sites in the municipality of Oiapoque, Amapá, Brazil.

This study was carried out in the municipality of Oiapoque (3° 49′ 29″ N, 51° 49′ 5″ W) and in the communities of Ilha Bela (3° 15′ 15.15″ N, 52° 15′ 24.54″ W) and Vila Brasil (3° 10′ 14.30″ N, 52° 19′ 48.69″ W), in the state of Amapá, in the far North of Brazil, on the border with French Guiana (Figure 1). French Guiana is an overseas department of France in South America, and its territory is largely covered by the Amazon rainforest. The soil rich in mineral resources attracts people, mainly Brazilians, who cross the border to access the various mining sites in remote areas of the Amazon rainforest in French Guiana. The present research used biological samples obtained primarily from two cross‐sectional studies to investigate the epidemiology of malaria in artisanal and small‐scale gold miners on the French‐Brazilian border, entitled “Study of the epidemiology of malaria in gold mines in Oiapoque” (Study 1 between 2018 and 2019) and “Radical Cure for Malaria among highly mobile and hard‐to‐reach populations in the Guiana Shield” (Study 2 in 2022). The investigation of *T. gondii* infection in this population was one of the secondary objectives of these studies, which justifies the use of this type of sample to identify exposure to this parasite.

### 2.2. Sampling and Study Population

Two cross‐sectional studies were carried out, including 289 participants in Study 1 and 191 in Study 2. Samples collected were used to test for *T. gondii* antibodies in 480 individuals who were enrolled at resting places in the municipality of Oiapoque. These sites are strategic locations for artisanal and small‐scale gold miners to rest, exchange gold, buy supplies, and receive medical care. Given the ethical challenges associated with conducting research among mobile and informal populations, particular care was taken to ensure that the consent process was clearly understood by all participants. The individuals who agreed to participate in the study signed a Free and Informed Consent Form containing detailed information about the objectives and procedures of the study. After identification of potential participants, miners were invited to participate and received a clear verbal explanation of the study procedures and objectives from the research team, regardless of their educational level, to ensure full comprehension. Participants were included in the study only after declaring that they were sufficiently informed and signing the informed consent form.

Given the difficult access to the study population, sampling was carried out by convenience, using the “opportunity to meet” method and an adaptation of the snowball method, according to Sadler et al. [21]. The population comprised individuals involved in gold mining activities in French Guiana, where this activity is informal, as well as people who lived or visited a mining site in French Guiana for various activities. To this end, this study included individuals of both sexes, over 18 years of age, who worked in mining areas in French Guiana, being at inclusion sites for less than 7 days before recruitment. It should be noted that although these individuals had different occupations at the mining sites, this population was referred to entirely as gold miners in a broad sense.

### 2.3. Biological Sample Collection

Five milliliters of whole blood was collected by venipuncture in Vacutainer tubes with EDTA. The samples were stored at −20°C in the Laboratório de Fronteiras in the municipality of Oiapoque and then transported frozen to the Laboratório de Doenças Parasitárias do Instituto Oswaldo Cruz (IOC) in Rio de Janeiro for initial malaria research. An aliquot of 1 mL was given to the Laboratório de Protozoologia (IOC) for serological and molecular detection of *T. gondii.*

### 2.4. Serological Processing

#### 2.4.1. Preparation of Blood Samples on Filter Paper

To test for anti‐*T. gondii* antibodies, the whole blood samples were applied to Whatman No. 3 cellulose filter paper, with a pore size of 6 μm and a diameter of 150 mm. It should be noted that the use of filter paper was chosen because obtaining plasma could have been compromised by freezing the blood samples immediately after collection, still in the municipality of Oiapoque. To prevent saturation of the filter paper impregnated with whole blood, according to Aston et al. [22], 50 μL of each blood sample was applied to a piece of filter paper and left to dry at room temperature for at least three hours. After drying, the blood‐impregnated filter papers were packed in ziplock bags with silica as a desiccant and stored at −20°C until processing.

#### 2.4.2. Serological Test

Anti‐*T. gondii* IgG and IgM antibodies were tested using the commercial kits Biolisa Toxoplasmosis IgG‐Bioclin (sensitivity: 98.38% and specificity: 98.70%) and Biolisa Toxoplasmosis IgM‐Bioclin (sensitivity: > 99.9% and specificity: 97%), following the manufacturer’s recommendations. The choice of this commercial kit was based on the manufacturer’s guarantee of its applicability for detecting antibodies against *T. gondii* using blood samples collected on filter paper. The plates were read using a Biolisa Reader‐R792 microplate reader configured with a 450‐nm and 650‐nm dual‐wavelength filter. Positivity was established by calculating the reactivity threshold (cut‐off) provided by the manufacturer for both IgG and IgM, as well as the reference values.

### 2.5. Molecular Analysis

Considering the low molecular positivity rates for *T. gondii* detected in blood samples or blood products from other human populations, the criteria were pre‐established for the inclusion of whole blood samples in molecular research on the protozoan, as in other studies [23–25]. In this context, processing all 480 samples could represent a substantial financial and time burden. Therefore, to enable the molecular detection of the protozoan, 100 whole blood samples that met the following criteria were selected for analysis: (i) samples that were seropositive for IgM detected by ELISA and (ii) IgG‐positive samples with the highest absorbance values. For this selection, the absorbance values of the samples were filtered in the descending order and, from these, a quantity representing 20% of the total samples was selected.

DNA was extracted from whole blood samples using the commercial kit Biospin/Blood/Cell/Tissue Genomic DNA Extraction Kit (Hangzhou Bioer 26 Technology), following the manufacturer’s protocol. The success of the extraction was confirmed by the amplification of the mammalian glyceraldehyde 3‐phosphate dehydrogenase (GAPDH) protein gene using a conventional PCR model, according to Birkenheuer et al. [26].

For the molecular detection of *T. gondii*, the extracted samples were subjected to nested polymerase chain reaction (nPCR) for the amplification of the GRA7 gene, using the primers GRA7FE/GRA7RE, for the amplification of a 322‐bp product, and GRA7FI/GRA7RI, for a 222‐bp product, according to Costa et al. [27].

Each reaction was carried out in a final volume of 50  μL containing 36.5  μL of ultrapure water, 1  μL of dNTP (10 mM), 1  μL of the primers GRA7FE/GRA7RE (first step) and GRA7FI/GRA7RI (second step), 5  μL of 10X buffer, 0.5  μL of Cellco Biotec Taq DNA polymerase, and 5  μL of the extracted DNA. The PCR mixes were subjected to an initial denaturation of 94°C for 2 min, 30 cycles of 94°C for 40 s (denaturation), 59°C for 40 s (annealing), 63°C for 40 s (extension), and a final extension of 63°C for 3 min. Aliquots of DNA from tachyzoites of the RH strain of *T. gondii*, maintained in Swiss Webster mice, were used as a positive control, while aliquots of ultrapure water served as a negative control. The nPCR products were visualized on a 1% agarose gel, stained with a solution of Gel Red Nucleic Acid Gel Stain (Biotium) and bromophenol blue, using a transilluminator with an ultraviolet lamp. The size of the expected bands was 322‐bp in the primary PCR and 222‐bp in the secondary PCR.

### 2.6. Epidemiologic Data

In parallel with the collection of blood samples, the participants were interviewed using a structured questionnaire covering sociodemographic information and characteristics of the mining sites at the resting places. This questionnaire was used to characterize this population and identify their main health problems [28]. The 191 participants recruited in 2022 were also asked questions about their lifestyle, such as their main water sources and consumption of game meat.

### 2.7. Variables

For the analysis of the type of mining, we considered the alluvial type, which is usually carried out in riverbeds and streams; the pit type, using excavation; and/or both (alluvial and pit) and mining with the aid of machines, which are installed on rafts and churn up the riverbeds.

### 2.8. Statistical Analysis

The results of the serodiagnosis and the variables were analyzed using the statistical program Epi Info 7.2.5.0 (CDC, Atlanta, USA). Initially, a univariable exploratory analysis of the data was carried out using Pearson’s chi‐square and Fischer’s exact tests, with a significance level of 5%. To avoid excluding potentially important variables in an exploratory study, variables with *p* values ≤ 0.2 were subjected to multivariable analysis using a logistic regression model. Finally, the odds ratios were estimated with their respective 95% confidence intervals. Associations with *p* values < 0.05 were considered significant.

### 2.9. Ethics Statement

The two projects were submitted to the CEP/Conep system; Project 1 (CAAE 80251017.8.0000.5248) was approved under number 5.799.042, and Project 2 (54888021.5.0000.5248) under number 6.298.356.

## 3. Results

Out of 480 gold miners, most were men (84%), 92% were between 21 and 60 years old, and 89.5% came from the states of Maranhão, Amapá, and Pará (Table 1). The prevalence of IgM and IgG anti‐*T. gondii* antibodies was 0.8% (*n* = 4/480, 95% CI = 0.3–2.1) and 79.4% (*n* = 381/480, 95% CI = 75.5–82.8), respectively. All the IgM‐positive samples were positive for IgG. The population of gold miners studied was composed of Brazilians. Seroprevalence was higher in males (80.1%), in individuals aged between 41 and 60 (81.2%), born in the Brazilian state of Amapá (84%), who had been working: in mining areas for 10 or more years (81.6%), in machine‐type mines (89.5%). Among the occupations reported by the participants in this study, the seroprevalence of *T. gondii* was higher among itinerant vendors (85.4%) than among other occupations.

**TABLE 1:** Serological results for IgG anti‐*T. gondii* and univariable analysis according to sociodemographic data and work in mining in the population of miners in the municipality of Oiapoque/Amapá, 2018–2022.

Variables	No. of samples	Positive		*p* value
		*N*	%	
*Sex*				
Male	402	322	80.10	0.46^a^
Female	78	59	75.64	

*Age group*				
< 20	20	15	75.00	0.09^b^
21–40	250	200	80.00	
41–60	192	156	81.25	
≥ 61	15	8	53.33	

*State of origin*				
Amapá	94	79	84.04	0.47^a^
Maranhão	227	181	79.74	
Pará	104	78	75.00	
Other	53	42	79.25	

*Years worked in the mines*				
≥ 10	245	200	81.63	0.27^a^
≤ 9	232	179	77.16	

*Type of mining*				
Alluvial	227	172	75.77	0.19^b^
Machine	48	43	89.58	
Alluvial and pit	59	47	79.66	
Pit	72	57	79.17	
Other	66	56	84.85	

*Work activities*				
Gold miners	180	139	77.22	0.22^a^
Vendors	117	100	85.47	
Domestic workers/cookers	48	35	72.92	
Others	133	106	79.70	

*Female of childbearing age*				
Yes	48	37	77.08	0.85^a^
No	29	21	72.71	

^a^Chi‐squared test.

^b^Fisher’s exact test.

Among female participants, seropositivity for antibodies against *T. gondii* was more frequent among women of childbearing age (14–40 years) (77%) than those outside this age group. There was no evidence of an association between this variable and seropositivity (*p* = 0.76). During enrollment, two participants reported being pregnant, only one of whom was reactive for IgG against *T. gondii*.

It was possible to obtain information on associated factors within the mining area during Study 2 (Table 2). Based on the univariable exploratory analysis, the following variables were selected for multivariate analysis: “age group” and “type of mining” (Table 3), and the variables “consumption of game meat in the last month” and consumption of “peccary” and “armadillo” meat (Table 4). There was no statistically significant difference in the logistic regression model for the sociodemographic variables and the mining characteristics (Table 3) and for the variables “consumption of game meat in the last month,” “consumption of peccary meat,” and “consumption of armadillo meat” (Table 4).

**TABLE 2:** Serological results for anti‐*T. gondii* IgG and univariable analysis according to lifestyle data in the population of miners in the municipality of Oiapoque/Amapá, 2022.

Source of water	Variables	No. of samples	Positive		*p* value
			*N*	%	
Well	Yes	119	99	83.19	0.73^a^
	No	72	62	86.11	

Surface water (e.g., stream, river, standing water)	Yes	161	135	83.85	1.0^b^
	No	30	26	86.67	

Rainwater harvesting	Yes	1	1	100	1.0^b^
	No	190	160	84.21	

Other	Yes	2	2	100	1.0^b^
	No	189	159	84.13	

Water treatment	No	168	142	84.52	0.7^b^
	Yes, sometimes	23	19	82.61	

Consumption of game meat in the last month	Yes	135	109	80	0.03^b^
	No	46	44	95.65	
	Doesn’t know/didn’t want to answer	10	8	80	

Consumption of capybara/paca/cutia meat	Yes	73	63	86.30	0.69^a^
	No	118	98	83.05	

Consumption of peccary meat	Yes	110	87	79.09	0.02^b^
	No	81	74	91.36	

Consumption of armadillo meat	Yes	16	11	68.75	0.14^b^
	No	175	150	85.71	

Consumption of wild bird meat	Yes	4	3	75	0.49^b^
	No	187	158	84.49	

Consumption of alligator/lizard/turtle meat	Yes	1	1	100	1.0^b^
	No	190	160	84.21	

Consumption of tapir meat	Yes	2	2	100	1.0^b^
	No	189	159	84.13	

Consumption of deer meat	Yes	2	1	50	0.29^b^
	No	189	160	84.66	

Presence of cats	Yes	10	1	90	0.99^b^
	No	181	151	83,4	

^a^Chi‐squared test.

^b^Fisher’s exact test.

**TABLE 3:** Multivariable logistic regression model for sociodemographic variables and mining characteristics with *p* value ≤ 0.2 analyzed in the population of miners in the municipality of Oiapoque/Amapá, 2018–2022.

Variable (*n* = 480)	Multivariate logistic regression				
	Coefficient	Standard error	Degrees of freedom	*p* value	Odds ratio
Age group	−0.1392	0.1824	6	0.4454	0.8700 (0.6085–1.2440)
Alluvial	0.3035	0.8551	6	0.7227	1.3546 (0.2535–7.2393)
Machine	1.3021	0.9652	6	0.1773	3.6769 (0.5545–24.3837)
Alluvial and pit	0.4846	0.9007	6	0.5905	1.6235 (0.2779–9.4863)
Pit	0.4751	0.8890	6	0.5930	1.6082 (0.2816–9.1839)
Other	0.8784	0.9096	6	0.3342	2.4070 (0.4048–14.3137)

**TABLE 4:** Multivariable logistic regression model for lifestyle variables with *p* value ≤ 0.2 analyzed in the population of miners in the municipality of Oiapoque/Amapá, 2022.

Variable (*n* = 191)	Multivariate logistic regression				
	Coefficient	Standard error	Degrees of freedom	*p* value	Odds ratio
Consumption of game meat in the last month	0.5449	0.3353	3	0.1042	1.7244 (0.8938–3.3271)
Consumption of peccary meat	0.9154	0.479	3	0.0539	2.4977 (0.9847–6.3355)
Consumption of armadillo meat	0.8007	0.591	3	0.1761	2.2271 (0.6981–7.1044)

Out of the total number of samples, 100 were subjected to molecular analysis. Of these, 2% showed products compatible with 222‐bp fragments of the GRA7 gene in nPCR, which were only detected in secondary PCR. The samples that were positive in the molecular analysis were also positive in the serological analysis, only for IgG antibodies.

## 4. Discussion

The present study detected high *T. gondii* seroprevalence in a population of artisanal gold miners from the Brazilian Amazon region. Regional comparisons with the literature were limited, as no epidemiological surveys were recovered that evaluated serological positivity for *T. gondii* in populations with a similar occupational profile. Lower seroprevalences were detected in coal miners sampled in China (7.7%), Ukraine (37.7%), and Mexico (60%) [29–31]. However, comparisons between the seroprevalence values observed in this study and those described in the literature should be interpreted carefully. Factors such as the type of population—occupational profiles in particular, environmental factors, sampling methodology, inclusion criteria, serological tests used, and their respective established cut‐off points may yield differences in seropositivity for anti‐*T. gondii* antibodies. The seroprevalence observed may not fully represent the true exposure to *T. gondii* among this population in the region, as one of the reasons miners sought rest areas was to obtain medical care. This may suggest a greater concern for health among these individuals compared with miners who rarely leave the mining sites. This epidemiological investigation was the first to demonstrate exposure to *T. gondii* in the population of Brazilian miners working informally on the French–Brazilian border.

Seropositivity for *T. gondii* in the population studied was similar to the frequencies of seropositivity for antibodies against the parasite reported in other populations in the Brazilian Amazon region, with different levels of contact with the forest, such as in urban populations, 81.9%; in rural populations, 73.3%; and in indigenous communities, 73.5% [5, 6, 32]. The gold miners in this study come from different Brazilian states that are part of the Legal Amazon, especially Maranhão, Pará, and Amapá, so the high seropositivity reported here may have been due to the high seroprevalence of parasitosis in these Brazilian states, considering that one of the main limitations of this study is the inability to determine the moment and time of exposure to *T. gondii*. Therefore, the reported seroprevalence reflects, in part, previous exposure in these regions.

The serological test for *T. gondii* infection conducted in this investigation used whole blood on filter paper as the biological sample. Testing for anti‐*T. gondii* antibodies in biological samples on filter paper has already been carried out on human and animal specimens [33, 34]. Given the availability of this type of biological sample to test for anti‐*T. gondii* antibodies in this study, the serological method chosen was enzyme immunoassay. The simple collection, storage, and transportation of this type of biological sample, together with its accessibility, sensitivity, and specificity, make it an important tool for the surveillance of infectious diseases in highly mobile populations in hard‐to‐reach areas [35, 36]. However, the use of dried blood spots on filter paper could present limitations, including variable sample volume, dilution during the elution process, and potential antibody degradation, which may affect the sensitivity of serological assays.

Records of the presence of domestic cats (*Felis catus*) at mining sites were obtained only from the second group of miners interviewed, and no significant association was observed between this variable and seropositivity for *T. gondii*. In this context, if it is assumed that miners were exposed to the parasite at the mining sites, it is reasonable to consider that other definitive hosts of the sylvatic cycle may be contributing to environmental contamination with oocysts [37]. However, the data obtained in the present study are not robust enough to support this hypothesis, as indicated by the low serological IgM and molecular positivity observed. Furthermore, as previously mentioned, contact with felids at mining sites may not necessarily be related to *T. gondii* seropositivity, since these individuals may have been exposed to the parasite prior to entering mining activities.

Although in the univariate analysis, the consumption of game meat, specifically peccary (*Tayassu* spp.), was associated with *T. gondii* infection, these associations were not maintained in the multivariate analysis, showing that it may be due to the chance or lack of power. However, Carme et al. [38] and de Thoisy et al. [39] demonstrated in their studies the high prevalence of *T. gondii* infection in wild animals in uninhabited regions of the French‐Guyanese Amazon, especially in peccary. The authors reinforce the role of these animals as reservoirs of *T. gondii* and a possible source of human infection in these areas through the consumption of their raw or undercooked meat. Additionally, a study by Vitalino et al. [40] identified atypical strains of the protozoan isolated from free‐living peccary in the Brazilian Amazon region that are phylogenetically grouped with strains originating in French Guiana. This evidence reinforces the importance of this wild species in maintaining the *T. gondii* cycle in the Amazonian environment and its possible involvement in the cases of severe acute toxoplasmosis historically reported in this region. While no association was found between game meat consumption and seropositivity in the study population, exposure to *T. gondii* cysts through dietary habits cannot be ruled out, as the participants included reported consuming this animal product.

The molecular investigation of *T. gondii* carried out in this study was done by PCR due to its greater sensitivity and the possibility that it could be used as a complement to the serological diagnosis of acute toxoplasmosis [41]. In addition, the nPCR model targeting the GRA7 gene allows not only greater sensitivity but also a suitable target for detecting DNA from clonal strains of the protozoan and also those considered atypical, such as those circulating in the region of this study [27, 42]. In general, the detection of *T. gondii* DNA in peripheral blood samples is associated with parasitemia caused by a possible acute and recent infection. Therefore, the choice of molecular testing for the parasite in this type of sample was due to the possibility of identifying evidence of recent infection, which would give more strength to the hypothesis of exposure to the protozoan at the mining sites.

In this study, 2% of the samples submitted for molecular diagnosis showed amplification of fragments compatible with the GRA7 gene of *T. gondii* using the nPCR model. It should be noted that the positive samples in the molecular analysis were obtained from individuals who reacted only to anti‐*T. gondii* IgG antibodies. The low frequency of positive samples in the PCR was already expected, considering the high IgG positivity associated with the low IgM positivity detected, possibly as a result of a chronic infection. In addition, the low molecular positivity in the PCR could be the result of DNA degradation due to the length of time the blood was collected and stored. It is important to highlight that the low molecular positivity observed may also be attributed to the limitations of the nPCR approach such as the increased risk of contamination due to the two amplification rounds, longer processing time, and dependence on DNA quality, which may compromise detection in samples with low parasite load.

Taken together, the association of positive IgG and molecular results may represent late parasitemia resulting from the intermittent release of bradyzoites in chronically infected individuals or from a possible reinfection event [43, 44]. These scenarios could explain the detection of circulating *T. gondii* DNA in chronically infected individuals in the absence of IgM antibodies against the parasite and other laboratory evidence of recent infection.

This study demonstrated for the first time the exposure of Brazilian artisanal gold miners to *T. gondii*, working in illegal mining sites in French Guiana, recruited at resting places in the far north of Brazil. Although no statistically significant association was found, higher seropositivity was detected in adult men who had been working in mining areas for more than 10 years and in machine‐type mines. Furthermore, the presence of circulating parasite DNA observed in this study still requires further investigation, considering the high genetic diversity of *T. gondii* in the region. This becomes even more relevant when considering population groups in areas that are hard to reach, such as Brazilian miners. Thus, future epidemiological surveys from a one health perspective associated with the implementation of prevention and control measures are necessary to identify other possible sources of infection specific to the epidemiological scenario of extraction sites, as well as to reduce the exposure of individuals working in these mining areas to *T. gondii*.

## 5. Limitations of the Study

Recruiting through the “opportunity to meet” method imposes limits on sample representativity. The need for the gold miners to travel to the resting places is a factor that reduced the possibility of increasing the sample number. The large number of categories in certain variables, together with the uneven distribution of individuals across these categories, may have limited the statistical power of the analysis. The questionnaire analysis may have been limited because the variables were originally designed for malaria studies, and those more relevant to *T. gondii* infection were collected only in the second study. The time interval between the two collection periods may reflect different epidemiological scenarios, thus acting as a confounding factor. Finally, regarding the inclusion criteria for samples for molecular analysis, additional methodological tools, such as the avidity test, could strengthen and expand the inclusion of samples for the detection of *T. gondii* DNA.

## Funding

This research was funded by the Coordination of Superior Level Staff Improvement (CAPES), Brazil, Finance code 001, the National Council for Scientific and Technological Development (CNPq), and the European Interregional Amazon Cooperation Program (IACP N Synergie 8754).

## Conflicts of Interest

The authors declare no conflicts of interest.

## Data Availability

The data that support the findings of this study are available from the corresponding author upon reasonable request.
